# Berufliche Tätigkeiten im Freien als Risikofaktor für Melanome der Gesichtshaut: UV‐Exposition, Risikowahrnehmung und berufliche Relevanz

**DOI:** 10.1111/ddg.15995_g

**Published:** 2026-09-08

**Authors:** Susanne Dugas‐Breit, Jessica C. Hassel, Martin Dugas, Hans‐Joachim Schulze

**Affiliations:** ^1^ Sektion Dermatoonkologie Hautklinik und Nationales Centrum für Tumorerkrankungen Universitätsklinikum Heidelberg; ^2^ Institut für Medizinische Informatik Universitätsklinikum Heidelberg; ^3^ Abteilung für Dermatologie, Fachklinik Hornheide, Münster

**Keywords:** Berufliche UV‐Exposition, Melanom, präventive Maßnahmen, Risikowahrnehmung, Melanoma, occupational UV exposure, preventive measures, risk awareness

## Abstract

**Hintergrund:**

Diese Studie untersuchte UV‐Exposition und präventive Verhaltensweisen bei Melanompatienten, mit Fokus auf berufliche Tätigkeiten im Freien, Melanomcharakteristika, Risikowahrnehmung und Schutzverhalten.

**Patienten und Methodik:**

In dieser Querschnittsstudie wurden demographische Daten, Melanomcharakteristika, UV‐Exposition, Risikowahrnehmung und Präventionsmaßnahmen analysiert. Die Daten wurden nach beruflicher Exposition (Innenraum vs. im Freien) stratifiziert und mittels logistischer Regression auf Prädiktoren der Melanomlokalisation untersucht.

**Ergebnisse:**

Von 406 Patienten (54% weiblich; medianes Alter 57 Jahre) hatten 59 (15%) eine Outdoor‐Arbeit ausgeübt. Diese hatten häufiger Melanome in sonnenexponierten Körperregionen und eine höhere Prävalenz von Melanomen der Gesichtshaut (p = 0,020). Betroffene mit Melanomen der Gesichtshaut hatten doppelt so lange im Freien gearbeitet als jene mit Melanomen an anderen Lokalisationen, was auf eine Dosis‐Wirkungs‐Beziehung hinweist. In der logistischen Regression waren Outdoor‐Arbeit (OR 2,47) und Alter (OR 1,05) signifikante Prädiktoren. 229 (57%) Patienten gaben an, vor der Diagnose nicht über schädlichen Wirkungen von UV‐Strahlung informiert gewesen zu sein. Von den Outdoor‐Arbeitern nutzten nur 3 (5%) häufig Sonnenschutz, während 33 (56%) diesen selten oder nie durchführten.

**Schlussfolgerungen:**

Outdoor‐Arbeit stellt in dieser Kohorte einen signifikanten Risikofaktor für Melanome der Gesichtshaut dar. Sonnenschutz und Risikowahrnehmung sind insbesondere bei im Freien Tätigen unzureichend, was die Notwendigkeit gezielter Präventionsmaßnahmen unterstreicht.

## EINLEITUNG

Solare ultraviolette (UV‐)Strahlung gilt als der bedeutendste umweltbedingte Risikofaktor für die Entstehung eines Melanoms. Die Inzidenz des Melanoms steigt weltweit an, wobei UV‐Strahlung sowohl aus natürlichen als auch aus künstlichen Quellen eine zentrale Rolle bei der Krankheitsentstehung spielt. Der Zusammenhang zwischen UV‐Exposition und dem Plattenepithelkarzinom der Haut ist gut belegt,^1^, ^2^ was 2015 in Deutschland zur Anerkennung des kutanen Plattenepithelkarzinoms als Folge beruflicher natürlicher UV‐Exposition als Berufskrankheit führte.^3^ Der Nachweis eines direkten Zusammenhangs zwischen beruflicher UV‐Exposition und der Entstehung von Melanomen ist hingegen bislang uneinheitlich.^4^, ^5^, ^6^, ^7^, ^8^ Diese Inkonsistenz ist unter anderem auf die multifaktorielle Pathogenese des Melanoms zurückzuführen, die genetische Prädisposition, intermittierende UV‐Exposition sowie chronische kumulative UV‐Schädigung umfasst.^9^, ^10^

Etwa 12% der Erwerbstätigen in Deutschland arbeiten ausschließlich oder überwiegend im Freien, zum Beispiel im Baugewerbe, in der Landwirtschaft, Forstwirtschaft, im Gartenbau oder in anderen Tätigkeitsfeldern im Freien.^11^ Rund 7,2 Millionen Beschäftigte verbringen regelmäßig mehr als 22% ihrer Arbeitszeit im Freien oder erhalten pro Jahr mehr als 150 Standard‐Erythem‐Dosen UV‐Strahlung.^12^ Die jährliche UV‐Exposition dieser Beschäftigten ist im Durchschnitt bis zu dreimal höher als die von Beschäftigten ohne Tätigkeiten im Freien.^13^ Messungen von Wittlich zeigen, dass der von der WHO vorgeschlagene Grenzwert für berufliche UV‐Exposition regelmäßig um den Faktor fünf überschritten wird – ein Wert, der so bei keinem anderen Karzinogen erreicht wird.^14^, ^15^

Das konventionelle Klassifikationssystem für Melanome basiert auf histologischen Wachstumsmustern und unterteilt in vier Hauptsubtypen: superfiziell spreitend, Lentigo‐maligna‐Typ, nodulär und akrolentiginös.^16^, ^17^, ^18^ Der Subtyp des Lentigo‐maligna‐Melanoms (LMM), der 5%–15% aller Melanome ausmacht, tritt nahezu ausschließlich in chronisch sonnenexponierten Hautarealen auf.^19^, ^20^, ^21^, ^22^ Dies weist auf einen klaren Zusammenhang mit chronischer (kumulativer) UV‐Exposition hin. Dennoch gibt es bislang keine epidemiologischen Studien, die ausreichende Evidenz dafür liefern, dass eine bestimmte Bevölkerungsgruppe aufgrund beruflicher Exposition (chronische kumulative UV‐Strahlung) ein signifikant höheres Risiko für die Entwicklung eines LMM aufweist.^7^, ^13^, ^21^, ^22^

Trotz der bekannten Risiken werden präventive Maßnahmen unter im Freien tätigen Personen weiterhin unzureichend angewendet. Frühere Studien zeigten eine geringe Risikowahrnehmung in Bezug auf UV‐Strahlung und eine unzureichende Nutzung von Schutzmaßnahmen wie Sonnenschutzmitteln, Schutzkleidung oder Abschattung.^23^, ^24^, ^25^ Eine 2019 von der *Bundesanstalt für Arbeitsschutz und Arbeitsmedizin durchgeführte* Befragung ergab, dass nur ein Drittel der im Freien Tätigen in Deutschland regelmäßig über die Gefahren solarer Strahlung unterwiesen wurde.^11^ Zudem fehlten vor dieser Studie standardisierte Instrumente zur Erfassung der Risikowahrnehmung und des Sonnenschutzverhaltens in Bezug auf UV‐Exposition, insbesondere bei im Freien Tätigen.

Diese Studie zielt darauf ab, diese Lücken zu schließen, indem sie die berufliche UV‐Exposition, die Prävalenz und Charakteristika der Melanomsubtypen sowie die Risikowahrnehmung und Umsetzung präventiver Maßnahmen in einer Kohorte von Melanompatienten untersucht.

## PATIENTEN UND METHODIK

### Studiendesign

Es wurde eine monozentrische Querschnittsstudie in der Abteilung für Dermatologie der Fachklinik Hornheide durchgeführt. Eingeschlossen wurden Patientinnen und Patienten im Alter von ≥ 18 Jahren mit einem Melanom im Stadium 0 bis IV gemäß den AJCC‐Leitlinien von 2017.^26^ Ausgeschlossen wurden Personen ohne ausreichende deutsche Sprachkenntnisse oder solche, die die Fragebögen nicht selbstständig ausfüllen konnten. Zwischen 2019 und 2021 füllten die Teilnehmenden nach schriftlicher Einwilligung im Rahmen ihrer klinischen Routineuntersuchung zusätzlich einen elektronischen Fragebogen aus. Die Studie wurde im Deutschen Register für klinische Studien registriert (DRKS00016939).

### Erhebungsinstrumente und Datenmanagement

Der elektronische Fragebogen erfasste demografische Daten, den beruflichen Hintergrund, berufliche und private UV‐Exposition, Anzahl der Sonnenbrände in Kindheit und Erwachsenenalter, Risikowahrnehmung hinsichtlich UV‐Strahlung sowie präventives Verhalten. Der berufliche Hintergrund beinhaltete Angaben zum Wirtschaftszweig, zum aktuellen Beschäftigungsstatus (zum Beispiel Vollzeit, Teilzeit, im Ruhestand) sowie dazu, ob die Tätigkeit überwiegend in Innenräumen oder im Freien ausgeübt wurde.

Teilnehmende wurden als Outdoor‐Gruppe klassifiziert, wenn sie angaben, regelmäßig im Freien zu arbeiten oder gearbeitet zu haben. Alle übrigen wurden der Indoor‐Gruppe zugeordnet, unabhängig vom aktuellen Beschäftigungsstatus, sofern keine regelmäßige berufliche Tätigkeit im Freien vorlag. Zur Erfassung der Risikowahrnehmung in Bezug auf UV‐Strahlung wurde gefragt: „Wussten Sie (früher), dass Sonnenlicht schädlich sein kann?“ (Antwortoptionen: ja/nein). Dieses selbstberichtete Item diente der Operationalisierung der UV‐Risikowahrnehmung.

Alle Fragen nutzten vorgegebene kategoriale Antwortoptionen (zum Beispiel „nie“, „selten“, „manchmal“, „häufig“). Der Hauttyp nach Fitzpatrick wurde mittels standardisiertem Fragebogen erfasst.^27^ Der vollständige Fragebogen ist im Online‐Supplement enthalten. Die ausgewählten Fragen wurden aus etablierten Quellen zusammengestellt, darunter aus dem *Work Ability Index* sowie aus der bevölkerungsbasierte Fall‐Kontroll‐Studie FB‐181.^1^, ^28^

Zusätzlich wurden Angaben zu Subtyp, Lokalisation und Stadium des Melanoms nach der *International Classification of Diseases for Oncology* (ICD‐O‐3, 2. Auflage 2019) erhoben.^29^ Die Ergebnisse dieser Studie werden gemäß den STROBE‐Empfehlungen (*Strengthening the Reporting of Observational Studies in Epidemiology*) berichtet.^30^

### Statistik

Es handelt sich hier um eine Querschnittsstudie. Die elektronisch erfassten Studiendaten wurden auf Vollständigkeit und Plausibilität überprüft. Von 414 Patienten lieferten 406 auswertbare Daten (zur Fallzahlschätzung und zum STROBE‐Flowchart siehe Online‐Supplement). Es wurden deskriptive Analysen aller klinischen Parameter und der Fragebogendaten zu UV‐Exposition und beruflicher Tätigkeit durchgeführt. Die Auswertung erfolgte mit dem Statistikprogramm R (Version 4.1.4., ggplot2‐Bibliothek). Zusammenhänge zwischen Variablen wurden unter Anwendung zweiseitiger Signifikanztests (α = 0,05) untersucht. Fehlende Werte wurden ohne Imputation aus den jeweiligen Analysen ausgeschlossen.

Für kontinuierliche Daten wurden nichtparametrische Tests (ohne Annahme einer Normalverteilung) eingesetzt, insbesondere Wilcoxon‐Tests (ungepaart oder gepaart). Für kategoriale Daten wurden der Chi‐Quadrat‐Test nach Pearson oder der exakte Fisher‐Test verwendet.

Eine multivariable logistische Regression wurde durchgeführt, um die Signifikanz der folgenden 15 Prädiktorvariablen für das Auftreten von Melanomen der Gesichtshaut zu prüfen: Geschlecht, Alter, Body‐Mass‐Index, familiäre Melanomanamnese, Ausbildung, Tätigkeit im Freien vs. in Innenräumen, Sonnenschutz bei der Arbeit, künstliche UV‐Exposition (Freizeit/beruflich), Solariumnutzung, UV‐Exposition in der Freizeit, Sonnenschutz in der Freizeit, Sonnenbrände in der Kindheit, Sonnenbrände im Erwachsenenalter, Feldkanzerisierung sowie Hauttyp nach Fitzpatrick. Variablen mit einem p‐Wert < 0,05 in der univariablen logistischen Regression wurden in ein Forward‐Selection‐Verfahren aufgenommen, um das multivariable Modell zu erstellen. Eine Adjustierung für multiples Testen erfolgte nicht (nicht‐konfirmatorische Beobachtungsstudie).

## ETHIK

Diese Studie wurde in Übereinstimmung mit den Prinzipien der Deklaration von Helsinki durchgeführt. Die Genehmigung wurde von der Ethikkommission der Ärztekammer Westfalen‐Lippe erteilt (Nummer 2019‐258‐f‐S). Die in diesem Manuskript beschriebenen Patienten haben ihr schriftliches Einverständnis zur Veröffentlichung ihrer Falldetails gegeben.

## ERGEBNISSE

### Charakteristika und berufliche Situation der Patienten

Die Studienkohorte umfasste 406 Patienten im Alter von 21–89 Jahren, davon waren 219 (54%) weiblich. Weitere demografische und klinische Details sind in Tabelle 1 dargestellt. Die Melanom‐Charakteristika und ‐Stadien sind in Tabelle 2 aufgeführt. Die mediane Tumordicke (Breslow, AJCC 2017) betrug 1,2 mm (0,1–35 mm; IQR 0,7–2,4 mm).

**TABELLE 1:** Charakteristika der Patientenpopulation.^*^

	Total n (%)	Indoor	Outdoor	p‐Wert
Alle Patienten	406	343 (85%)	59 (15%)^**^	
Weiblich	219 (54%)	205 (60%)	12 (20%)	<0,001
Männlich	187 (46%)	138 (40%)	47 (80%)	
***Medianes Alter in Jahren (Spannweite)***				
Alle Patienten	57 (21‐89)	56	58	0,24
Weiblich	56 (22‐89)	54	57,5	
Männlich	58 (21‐89)	58	58	
***Hauttyp (Fitzpatrick)***				0,25
I	6 (2%)	6 (2%)	0 (0%)	
II	198 (51%)	172 (51%)	24 (41%)	
III	186 (45%)	152 (45%)	33 (56%)	
IV	9 (2%)	7 (2%)	2 (3%)	
V	0 (0%)	0 (0%)	0 (0%)	
VI	0 (0%)	0 (0%)	0 (0%)	
***Body‐Mass‐Index (BMI) (kg/m^2^)***				
Median (Spannweite)	26,9 (16,6‐47,8)	26,4	29,4	< 0,001
***Ausbildung*** ^**^				< 0,001
≤ 8 Jahre	148 (37%)	110 (32%)	36 (61%)	
10 Jahre	122 (30%)	111 (32%)	11 (19%)	
Abitur	90 (22%)	82 (24%)	8 (14%)	
Universitätsabschluss	44 (11%)	39 (11%)	4 (7%)	
***Arbeit***				0,001
Vollzeit	216 (53%)	174 (51%)	40 (68%)	
Teilzeit	80 (20%)	77 (22%)	3 (5%)	
Ohne Arbeit	28 (7%)	27 (8%)	1 (2%)	
Im Ruhestand	81 (20%)	65 (19%)	15 (25%)	
***Melanom in der Familienanamnese***				0,37
Nein	330 (82%)	275 (81%)	51 (86%)	
Ja	72 (18%)	64 (19%)	8 (14%)	

* Geringfügige Abweichungen von 100% sind auf Rundungsdifferenzen zurückzuführen.

** Vier Patienten machten keine Angaben hinsichtlich Indoor oder Outdoor‐Arbeit

**TABELLE 2:** Charakteristika der Melanome.

		Total n (%)	Indoor	Outdoor	p‐Wert
**Total**		406	343	59^*^	
***Typ***	Superfiziell spreitend	196 (48%)	167 (49%)	29 (49%)	0,92
	Nodulär	54 (13%)	44 (13%)	10 (17%)	0,44
	Lentigo maligna oder Lentigo maligna‐Melanom	48 (12%)	38 (11%)	7 (12%)	>0.99
	Akrolentiginös	18 (4%)	16 (5%)	1 (2%)	0,50
	Amelanotisch	5 (1%)	5 (1%)	0 (0%)	>0.99
	In‐situ‐Melanom	11 (3%)	10 (3%)	1 (2%)	>0.99
	Unspezifiziert oder andere	74 (18%)	63 (18%)	11 (19%)	>0.99
***Lokalisation***	Untere Extremität und Hüfte	111 (27%)	101 (29%)	10 (17%)	0,11
	Rumpf	132 (33%)	110 (32%)	22 (37%)	0,54
	Obere Extremität und Schulter	79 (19%)	69 (20%)	10 (17%)	0,75
	Gesicht	45 (11%)	31 (9%)	12 (20%)	0,033
	Kopf/Hals	26 (6%)	21 (6%)	3 (5%)	>0.99
	Unbekannt	11 (3%)	9 (3%)	2 (3%)	0,67
	Genital	2 (1%)	2 (1%)	0 (0%)	>0.99
***Melanom‐Stadium***	0	29 (7%)	22 (6%)	5 (8%)	0,81
	I	202 (50%)	182 (54%)	19 (32%)	0,03
	II	75 (19%)	58 (17%)	16 (27%)	0,16
	III	82 (20%)	66 (19%)	16 (27%)	0,29
	IV	14 (3%)	11 (3%)	3 (5%)	0,74

* Vier Patienten machten keine Angaben hinsichtlich Indoor oder Outdoor‐Arbeit

Insgesamt gaben 59 (15%) Patienten an, regelmäßig im Freien gearbeitet zu haben, mit einer medianen Dauer von 25 Jahren (3–52; IQR 14–40). Davon waren 47 (80%) männlich. In der Outdoor‐Gruppe befanden sich signifikant mehr Männer als in der Indoor‐Gruppe (n = 138; 40%; p < 0,001). Zudem zeigte die Outdoor‐Gruppe einen höheren Body‐Mass‐Index, eine geringere Ausbildung sowie einen höheren Anteil an Vollzeitbeschäftigung. Informationen zum angegebenen Wirtschaftszweig sind in der Tabelle S1 im Online‐Supplement aufgeführt.

### UV‐Exposition der Patienten

Angaben zur UV‐Exposition aller Patienten sind in Tabelle 3 zusammengefasst. Die Zeit im Freien während der Freizeit variierte; 78% der Befragten gaben an, sich (fast) täglich oder häufig im Freien aufzuhalten. Insgesamt berichteten 59% ein Solarium besucht zu haben; Sonnenbrände in Kindheit oder Erwachsenenalter waren häufig. Ein Anteil von 15 % der Befragten gab eine berufliche oder private Exposition gegenüber künstlicher UV‐Strahlung (zum Beispiel durch Schweißen) an. Eine Feldkanzerisierung sonnenexponierter Haut aufgrund chronischer UV‐Schädigung lag bei 12% der Patienten vor. Zwischen der Indoor‐ und der Outdoor‐Gruppe bestand diesbezüglich kein signifikanter Unterschied (p = 0,83).

**TABELLE 3:** Charakteristika der UV‐Exposition bei allen Patienten.^*^

	Total n (%)	Indoor	Outdoor	p‐Wert
***Zeit im Freien (Freizeit)***				
(Fast) täglich	115 (28%)	83 (24%)	31 (53%)	< 0,001
Oft	194 (48%)	170 (50%)	22 (38%)	0,23
Gelegentlich	88 (22%)	83 (24%)	5 (9%)	0,019
(Fast) nie	3 (1%)	3 (1%)	0 (0%)	>0.99
***Solarium‐Nutzung***				
Nie	166 (41%)	119 (35%)	43 (73%)	< 0,001
Wenige Male	160 (40%)	148 (43%)	12 (20%)	0,007
Einige Male	52 (13%)	49 (14%)	3 (5%)	0,08
(Fast) jährlich	4 (1%)	4 (1%)	0 (0%)	>0.99
Regelmäßig	24 (6%)	23 (7%)	1 (2%)	0,16
***Künstliche UV‐Exposition (beruflich)***				
Ja	11 (3%)	8 (2%)	3 (5%)	0,21
Nein	395 (97%)	332 (98%)	55 (95%)	
***Künstliche UV‐Exposition (privat)***				
Ja	49 (12%)	42 (12%)	6 (10%)	0,83
Nein	357 (88%)	298 (88%)	52 (90%)	
***Sonnenbrände in der Kindheit (< 18 Jahre)***				
Nie	24 (6%)	22 (6%)	2 (3%)	0,57
Wenige Male	168 (41%)	132 (39%)	34 (58%)	0,049
Einige Male	97 (24%)	89 (26%)	7 (12%)	0,042
(Fast) jährlich	77 (19%)	71 (21%)	6 (10%)	0,11
Mehrere pro Jahr	39 (10%)	28 (8%)	10 (17%)	0,06
***Sonnenbrände als Erwachsene***				
Nie	21 (5%)	17 (5%)	4 (7%)	0,54
Wenige Male	187 (46%)	158 (46%)	26 (44%)	0,92
Einige Male	120 (30%)	106 (31%)	14 (24%)	0,44
(Fast) jährlich	55 (14%)	48 (14%)	6 (10%)	0,57
Mehrere pro Jahr	22 (5%)	13 (4%)	9 (15%)	0,003
***Feldkanzerisierung***				
Ja	50 (12%)	42 (12%)	8 (14%)	0,83
Nein	355 (88%)	300 (88%)	51 (87%)	

* Geringfügige Abweichungen von 100% sind auf Rundungsdifferenzen zurückzuführen.

### Zusammenhang zwischen Melanomlokalisation und UV‐Exposition

Insgesamt gaben 84 (21%) Personen an, dass sich ihr Melanom in einer Körperregion befand, die stets dem Sonnenlicht ausgesetzt war, und 150 (37%) nannten eine Lokalisation, die je nach Jahreszeit häufig exponiert war. Bei 141 (35%) war die Lokalisation selten sonnenexponiert, bei 27 (7%) nie.

Eine Lokalisation im Gesicht war signifikant mit einer beruflichen Tätigkeit im Freien assoziiert (Odds Ratio 2,56; 95%‐Konfidenzintervall [KI] 1,12–5,57; p = 0,020), das heißt, Patienten der Outdoor‐Gruppe wiesen häufiger ein Melanom der Gesichtshaut auf als diejenigen der Indoor‐Gruppe (Tabelle 2).

Im Freien Tätige mit einem Melanom der Gesichtshaut hatten median 40 Jahre im Freien gearbeitet, während jene mit einem Melanom an anderer Stelle median 20 Jahre im Freien tätig waren (p = 0,015). In der Outdoor‐Gruppe trat ein Melanom signifikant häufiger in stets oder häufig sonnenexponierten Arealen auf (n = 39; 66%) als in der Indoor‐Gruppe (n = 192; 56%; p = 0,001). Diese Befunde sprechen für eine Dosis‐Wirkungs‐Beziehung für Melanome im Gesicht bei im Freien Tätigen.

Frauen wiesen signifikant häufiger ein Melanom an den unteren Extremitäten auf als Männer (n = 78; 70% vs. n = 33; 30%; p < 0,001). Dies erklärt den nichtsignifikanten Unterschied (p = 0,07) in der Verteilung dieser Lokalisation zwischen der Indoor‐ und Outdoor‐Gruppe (Tabelle 2). Zudem erhielten Frauen signifikant häufiger die Diagnose eines Melanoms im Stadium I als Männer (n = 123; 61% vs. n = 79; 39%; p = 0,003). Auch dies erklärt den signifikanten Unterschied (p = 0,004) in der Verteilung von Stadium I zwischen Indoor‐ und Outdoor‐Gruppe (Tabelle 2).

Bezüglich Freizeitaktivitäten im Freien zeigte sich kein signifikanter Zusammenhang zwischen Häufigkeit der UV‐Exposition und Melanomlokalisation (Fishers exakter Test p = 0,40) sowie keine monotone Korrelation (Spearman‐Korrelation 0,038; p = 0,45). Zwischen histologischem Melanomtyp und Tätigkeit im Freien beziehungsweise in Innenräumen bestand ebenfalls kein Zusammenhang (p = 0,98).

Die multivariable logistische Regression mit *Forward Selection* zur Identifizierung von Prädiktoren für Melanome der Gesichtshaut ergab, dass der Status „Tätigkeit im Freien“ (OR 2,47; 95%‐KI 1,13–5,15; p = 0,018) sowie das Alter (OR 1,05; 95%‐KI 1,02–1,07; p < 0,001) die einzigen beiden signifikanten Risikofaktoren für ein Melanom der Gesichtshaut darstellten. (Details der univariablen logistischen Regression siehe Tabelle S2 im Online‐Supplement)

### Sonnenschutzverhalten

Insgesamt gaben 125 (31%) Personen an, während der Freizeit häufig Sonnenschutz zu verwenden, 166 (41%) manchmal, 95 (24%) selten und 18 (5%) nie. In der Outdoor‐Gruppe berichteten 15 (25%), niemals Sonnenschutz (zum Beispiel Sonnenschutzmittel oder Schutzkleidung) durchzuführen, 18 (31%) selten, 23 (39%) manchmal und nur drei (5%) häufig.

Auf die Frage nach dem Wissen über mögliche schädliche Wirkungen von Sonnenlicht gaben 229 (57%) Patienten an, vor der Diagnose nicht darüber informiert gewesen zu sein. Zwischen Indoor‐ und Outdoor‐Gruppe zeigte sich diesbezüglich kein signifikanter Unterschied (p = 0,21).

## DISKUSSION

Diese Studie unterstreicht die bedeutende Rolle der beruflichen UV‐Exposition bei der Entstehung von Melanomen der Gesichtshaut und hebt deren Relevanz als Berufskrankheit hervor. Unsere Analyse von 406 Patienten zeigt, dass im Freien Tätige ein signifikant höheres Risiko haben, ein Melanom der Gesichtshaut zu entwickeln als in Innenräumen Tätige (OR 2,47). Im Jahr 2021 berichtete das Krebsregister Baden‐Württemberg eine Inzidenz von 8,1% für Melanome der Gesichtshaut bei allen Patienten,^31^ was der Inzidenz der Indoor‐Gruppe unserer Studienpopulation entspricht. Diese Ergebnisse stimmen mit früheren Untersuchungen überein, die einen starken Zusammenhang zwischen chronischer UV‐Exposition und Melanomen im Kopf‐Hals‐Bereich zeigten, welcher bei Tätigkeiten im Freien häufig dem Sonnenlicht ausgesetzt ist.^32^, ^33^, ^34^, ^35^, ^36^ Tatsächlich wurde ein erhöhtes Risiko in Berufen wie der Landwirtschaft^37^ oder der Bauwirtschaft^38^ beschrieben.

Darüber hinaus wurde eine deutliche Dosis‐Wirkungs‐Beziehung identifiziert: Im Vergleich zu in Innenräumen Tätigen entwickelten im Freien Tätige signifikant häufiger Melanome in stets oder häufig sonnenexponierten Arealen. Zudem hatten im Freien Tätige mit einem Melanom der Gesichtshaut doppelt so lange im Freien gearbeitet wie jene, die ein Melanom an anderer Lokalisation entwickelt hatten. Es konnte kein statistisch signifikanter Zusammenhang zwischen UV‐Exposition in der Freizeit und dem Auftreten von Melanomen der Gesichtshaut festgestellt werden, was darauf hindeutet, dass die berufliche UV‐Exposition einen größeren Einfluss auf das Risiko hat als die UV‐Exposition in der Freizeit. Während die meisten Studien Melanome nach histologischem Subtyp kategorisierten,^17^, ^39^, ^40^ sollte künftige Forschung verstärkt anatomisch‐lokalisationsspezifische Risiken betrachten. In unserer Kohorte fanden wir keine statistisch signifikante Häufung von Feldkanzerisierung als Zeichen langfristiger chronischer UV‐Exposition in der Outdoor‐Gruppe im Vergleich zur Indoor‐Gruppe. Dies deckt sich mit unterschiedlichen Mutationsprofilen von Melanomen an chronisch sonnenexponierter gegenüber intermittierend exponierter Haut^9^, ^36^ und mit der Annahme anderer Autoren, dass unterschiedliche pathogenetische Wege in Abhängigkeit von der anatomischen Lokalisation existieren könnten.^41^, ^42^

Hinsichtlich der Umsetzung von Präventionsmaßnahmen zeigte diese Studie einen alarmierenden Mangel an Strategien zum Sonnenschutz am Arbeitsplatz: Trotz höherer UV‐Exposition berichteten nur 5% der Outdoor‐Gruppe über regelmäßige Anwendung von Sonnenschutzmaßnahmen während der Arbeit, während 56% diese nie oder selten nutzten. Im Vergleich dazu war die Verwendung von Sonnenschutzmitteln im Freizeitbereich unter unseren Patienten häufiger, was den Bedarf an gezielten Aufklärungsinitiativen im beruflichen Umfeld unterstreicht. Diese Ergebnisse stimmen mit früheren Studien überein,^43^ wonach sich 70% der im Freien Tätigen unzureichend vor UV‐Exposition geschützt fühlten.

Ob Melanompatienten häufiger eine Vorgeschichte von Sonnenbränden aufweisen als die Allgemeinbevölkerung, lässt sich mangels vergleichbarer Daten schwer beurteilen. In unserer Kohorte ähnelte die Häufigkeit von Sonnenbränden der in der Allgemeinbevölkerung: In einer Befragung aus dem Jahr 2016 gaben 41% der Erwachsenen an, im Vorjahr mindestens einen Sonnenbrand gehabt zu haben.^44^ Hinsichtlich der Nutzung von Solarien zeigte unsere Patientenkohorte jedoch eine deutlich höhere Prävalenz als die Allgemeinbevölkerung: Während 27% unserer Patienten von häufigen oder regelmäßigen Solariumbesuchen berichteten, waren es in einer Befragung aus dem Jahr 2007 nur 9%.^45^ Neuere Daten aus einer repräsentativen deutschen Bevölkerungsstudie aus den Jahren 2015–2022 zeigen einen weiteren Rückgang, mit einer aktuellen Nutzungsrate von 5%–6% in der Allgemeinbevölkerung.^46^ Trotz Fortschritten in der Prävention, einschließlich Verhaltensinterventionen und Sonnenschutzkampagnen,^47^, ^48^ bleibt die Umsetzung von Sonnenschutzmaßnahmen insgesamt unzureichend.

Unsere Ergebnisse decken zudem eine erhebliche Wissenslücke auf: 57% aller Patienten (229 von 406) gaben an, vor ihrer Melanomdiagnose nicht gewusst zu haben, dass Sonnenlicht schädlich sein kann. Daraus ergibt sich ein dringender Bedarf für kontinuierliche Aufklärung und Schulung in diesem Bereich, insbesondere im Rahmen der Berufsausbildung und am Arbeitsplatz.

In den letzten Jahren haben sich die Bemühungen zur Behebung dieser Defizite verstärkt. Insbesondere die Entwicklung standardisierter Messinstrumente wie des *Sun Exposure and Protection Index* ^49^ und der *Skin Cancer and Sun Knowledge Scale* ^50^ bietet wertvolle Werkzeuge zur Erfassung von Sonnenschutzverhalten und ‐wissen. Diese Instrumente sollten zusammen mit validierten, übersetzten Fragebögen^51^ verstärkt im beruflichen Kontext eingesetzt werden, um Risikoverhalten zu identifizieren und maßgeschneiderte Präventionsstrategien zu ermöglichen.

Unsere Studie weist mehrere Stärken auf: Sie basiert auf einer Kohorte relevanter Größe mit hohem Anteil vollständiger Datensätze, die umfassende Patienteninformationen und standardisierte medizinische Untersuchungen einschließt. Mehrere Befunde aus Krebsregisterdaten (zum Beispiel häufigere Lokalisation von Melanomen an den Beinen bei Frauen) spiegeln sich in unseren Ergebnissen wider, was die Qualität und Validität der Daten zusätzlich stützt.

Bei der Interpretation der Ergebnisse müssen jedoch Einschränkungen berücksichtigt werden: Es handelt sich um eine monozentrische Kohortenstudie mit Querschnittsdaten, weshalb die Ergebnisse möglicherweise nicht vollständig generalisierbar sind. Dosimetrische Daten zur beruflichen und freizeitbedingten UV‐Exposition liegen nicht vor. Die Bewertung beruht daher auf Patientenangaben, was die Genauigkeit der Expositions‐Risiko‐Korrelationen einschränken kann. Darüber hinaus wurde der in dieser Studie verwendete kombinierte Fragebogen nicht als Ganzes formell validiert, was eine methodische Limitation darstellt. Er basierte jedoch auf zuvor angewandten und in Fachzeitschriften begutachteten Instrumenten, wie dem *Work Ability Index* und der bevölkerungsbasierten FB‐181‐Studie.^1^, ^28^ Ein weiteres Problem ist ein Selektionsbias, da nur Patienten mit höheren Tumorstadien häufiger regelmäßig zur medizinischen Nachsorge erscheinen und in unserer Befragung daher leicht überrepräsentiert waren.^52^

Zusammenfassend traten Melanome der Gesichtshaut häufiger bei beruflich im Freien Tätigen auf und waren mit einer längeren Dauer beruflicher Sonnenexposition assoziiert, was auf eine potenzielle berufliche Relevanz ähnlich wie bei Plattenepithelkarzinomen der Haut hinweist. Es wurden erhebliche Defizite im Wissen und Verhalten zum UV‐Schutz festgestellt, was den anhaltend dringenden Bedarf unterstreicht, die Aufklärung über UV‐Schutzmaßnahmen in die Berufsausbildung aller im Freien tätigen Berufsgruppen einzubeziehen – insbesondere in Zeiten zunehmender UV‐Belastung.^53^ Zur Sicherstellung der Umsetzung sollten regelmäßige Überprüfungen fester Bestandteil der beruflichen Tätigkeit sein.

## DANKSAGUNG

Open access Veröffentlichung ermöglicht und organisiert durch Projekt DEAL.

## FINANZIERUNG

Die Studie wurde durch einen Zuschuss der Deutschen Rentenversicherung gefördert (Förderkennzeichen 622‐4023). Der Fördergeber hatte keinen Einfluss auf Studiendesign, Datenerhebung, Datenanalyse oder Berichterstattung dieser Studie.

## INTERESSENKONFLIKT

J.C.H. erhält Honorare von BMS, Immunocore, MSD, Novartis, Philogen, Pierre Fabre und Sanofi als Vortragende sowie von Onkowissen und Sunpharma als Mitglied von Advisory Boards. S.D.‐B., M.D. und H.‐J.S. erklären, dass kein Interessenkonflikt besteht.

**Ethikvotum**: Diese Studie wurde in Übereinstimmung mit den Prinzipien der Deklaration von Helsinki durchgeführt. Die Genehmigung wurde von der Ethikkommission der Ärztekammer Westfalen‐Lippe erteilt (Nummer: 2019‐258‐f‐S).

**Ethikerklärung**: Die in diesem Manuskript beschriebenen Patientinnen und Patienten haben ihr schriftliches Einverständnis zur Veröffentlichung ihrer Falldetails gegeben.

## Supporting information



Supplementary information
