# Supplementary material for: Berufliche Tätigkeiten im Freien als Risikofaktor für Melanome der Gesichtshaut: UV‐Exposition, Risikowahrnehmung und berufliche Relevanz
Source: J Dtsch Dermatol Ges. 2026 Sep 8;24(9):1232–40. [Article in German] doi: 10.1111/ddg.15995_g (PMC13551284; doi:10.1111/ddg.15995_g)
Supplement: Supplementary file 1 [file DDG-24-1232-s001.docx]

**Online Supplement**

Berufliche Tätigkeiten im Freien als Risikofaktor für Melanome der Gesichtshaut:
UV-Exposition, Risikowahrnehmung und berufliche Relevanz

Susanne Dugas-Breit, Jessica C. Hassel, Martin Dugas^*^, Hans-Joachim Schulze^*^

* Diese Autoren teilen sich die Letztautorenschaft.

Korrespondenzanschrift:
Susanne Dugas-Breit
Sektion Dermatoonkologie, Hautklinik und Nationales Centrum für Tumorerkrankungen, Universitätsklinikum Heidelberg, Heidelberg, Deutschland
susanne.dugas-breit@med.uni-heidelberg.de

**Fallzahlschätzung**

Die Fallzahl wurde unter folgenden Annahmen berechnet:
Es sollten 15 Prädiktoren mittels logistischer Regression analysiert werden, mit 10 Ereignissen pro Prädiktor und einer Ereignisrate von 0,4.
Daraus ergibt sich eine erforderliche Fallzahl von 375 Patientinnen und Patienten. Um bis zu 10 % unvollständige Datensätze zu berücksichtigen, wurde eine Gesamtkohorte von 413 Patientinnen und Patienten eingeplant.

**STROBE Flow Chart**

Auf Einschlussfähigkeit geprüft: n=414

Nicht bereit, den Fragebogen auszufüllen:
- aus Zeitgründen: n=4
- ohne Angabe von Gründen: n=2

Einwilligung erteilt: n=408

Fragebogen doppelt ausgefüllt: n=2

Vollständiger Datensatz: n=406

**Tabelle S1** Wirtschaftszweig, in dem die Patienten beschäftigt sind / waren

| **Wirtschaftszweig** | **Anzahl der Patienten** |
| --- | --- |
| Land-, Forstwirtschaft und Fischerei | 50 (12%) |
| Verarbeitendes Gewerbe ohne Baugewerbe | 29 (7%) |
| Baugewerbe | 3 (1%) |
| Handel, Gastronomie, Transport | 87 (21%) |
| Finanzwesen, Immobilien, unternehmensnahe Dienstleistungen | 22 (5%) |
| Öffentliche und private Dienstleistungen | 178 (44%) |
| Sonstige | 37 (9%) |

**Tabelle S2** Univariable logistische Regressionsanalyse für den Zusammenhang zwischen jedem einzelnen Prädiktor und dem Outcome „Melanom der Gesichtshaut“ (ohne Adjustierung für andere Prädiktoren). Die Odds Ratios (OR) werden zusammen mit den 95 %-Konfidenzintervallen (KI) sowie den zugehörigen p-Werten dargestellt.

| Variable | OR | 95% CI OR | p-Wert |
| --- | --- | --- | --- |
| Geschlecht | 1.19 | 0.639 – 2.26 | 0.584 |
| Alter | 1.05 | 1.02 – 1.07 | <0.001* |
| BMI | 0.985 | 0.925 – 1.04 | 0.630 |
| Melanom in der Familienanamnese | 1.17 | 0.506 – 2.45 | 0.698 |
| Ausbildung | 0.349 | 0.134 – 0.809 | 0.020* |
| Outdoor-Arbeit | 2.57 | 1.20 – 5.25 | 0.012* |
| Sonnenschutz bei der Arbeit | 0.421 | 0.086 – 1.93 | 0.265 |
| Künstliche UV Exposition | 0.669 | 0.194 – 1.76 | 0.463 |
| Solarium Nutzung | 0.546 | 0.260 – 1.10 | 0.099 |
| Zeit im Freien (Freizeit) | 1.48 | 0.656 – 3.38 | 0.340 |
| Sonnenschutz in der Freizeit | 0.399 | 0.126 – 1.53 | 0.140 |
| Sonnenbrände in der Kindheit (<18 Jahre) | 0.423 | 0.110 – 1.79 | 0.214 |
| Sonnenbrände als Erwachsene | 1.76 | 0.374 – 9.69 | 0.480 |
| Feldkanzerisierung | 0.514 | 0.239 – 1.20 | 0.103 |
| Hauttyp | 0.825 | 0.431 – 1.56 | 0.554 |
|  |  |  |  |

**Fragebogen**

**Wie groß sind Sie?**

__________ (in cm)

**Wie schwer sind Sie?**

__________ (in kg)

**Ist in Ihrer Familie ein Melanom aufgetreten?**

	Ja

	Nein

**Welchen Schulabschluss haben Sie?**

□ Noch in der Schule

□ Kein Schulabschluss

□ Volks-/Hauptschulabschluss/ 8. Klasse POS

□ Realschulabschluss/ mittlere Reife/ 10. Klasse POS

□ Fachabitur (Fachhochschulreife)

□ Abitur/ Hochschulreife/ EOS

□ Abgeschlossenes Hochschulstudium

□ Anderer Schulabschluss

**Welche der folgenden Angaben trifft auf Ihre Erwerbssituation
*vor dem Zeitpunkt* des Melanoms zu?**

 Ganztägig berufstätig

 Mindestens halbtags berufstätig

 Weniger als halbtags berufstätig

 RentnerIn wegen Erwerbsminderung/Alter

 Hausfrau/-mann

 Aus anderen Gründen nicht erwerbstätig

 Arbeitslos/erwerbslos

**Welchem Wirtschaftszweig lässt sich Ihre (frühere)Tätigkeit zuordnen? (Falls Sie mehrere Berufe hatten, wählen Sie bitte den Wirtschaftszweig, in dem Sie am längsten tätig waren.)**

 Land- und Forstwirtschaft

 Fischerei und Fischzucht

 Bergbau und Gewinnung von Steinen und Erden

 Verarbeitendes Gewerbe

 Energie- und Wasserversorgung

 Baugewerbe

 Handel; Instandhaltung und Reparatur von Kraftfahrzeugen und Gebrauchsgütern

 Gastgewerbe

 Verkehr und Nachrichtenübermittlung

 Kredit und Versicherungsgewerbe

 Immobilien, Vermietung, Dienstleistung für Unternehmen

 Öffentliche Verwaltung, Verteidigung, Sozialversicherung

 Erziehung und Unterricht

 Gesundheits-, Veterinär- und Sozialwesen

 Sonstige öffentliche und private Dienstleistungen

 Private Haushalte

 Exterritoriale Organisationen und Körperschaften

 Produzierendes Gewerbe o.n.A. bzw. nicht zuzuordnen

 Dienstleistungen o.n.A. bzw. nicht zuzuordnen

 Nicht zuzuordnen

**Haben Sie beruflich regelmäßig im Freien gearbeitet (z.B. als Gärtner, Bauarbeiter o.ä.)?**

 Ja

 Nein

***wenn ja:* Wie viele Jahre haben Sie beruflich regelmäßig im Freien gearbeitet?**

____ Jahre

***wenn ja:* Haben Sie *beruflich* Sonnenschutz (z.B. Sonnencreme, Sonnenschutzkleidung)
angewendet?**

 Nie

 Selten

 Manchmal

 Häufig

**Waren Sie *künstlicher* UV-Strahlung ausgesetzt?**

 Ja, beruflich (z.B. durch Schweißen)

 Ja, privat (durch Hobbys)

 Nein

**Waren Sie schon mal in einem Solarium?**

 Nie

 Wenige Male

 Mehrere Male

 (Fast) jährlich

 Regelmäßig

**Befindet sich Ihr Melanom in einer Körperregion, die der Sonnenstrahlung ausgesetzt war?**

 Immer

 Je nach Saison oft

 Selten

 Nie

**Wie würden Sie Ihr Freizeitverhalten im Freien einschätzen?**

 (Fast) täglich draußen

 Oft draußen

 Ab und zu draußen

 (Fast) nie draußen

**Haben Sie in der Freizeit Sonnenschutz (z.B. Sonnencreme, Sonnenschutzkleidung)
angewendet?**

 Nie

 Selten

 Manchmal

 Häufig

**Wie viele Sonnenbrände (auch „nur“ Rötung der Haut) hatten Sie VOR dem 18. Lebensjahr?**

 Nie

 Einzelne

 (Fast) jährlich

 Mehrere jährlich

**Wie viele Sonnenbrände (auch „nur“ Rötung der Haut) hatten Sie NACH dem 18. Lebensjahr?**

 Nie

 Einzelne

 (Fast) jährlich

 Mehrere jährlich

**War Ihnen (früher) bewusst, dass Sonnenstrahlung schädlich sein kann?**

	Ja

	Nein

**Wie ist Ihr allgemeiner Gesundheitszustand?**

 Ausgezeichnet

 Sehr gut

 Gut

 Weniger gut

 Schlecht

**Nun noch einige Fragen zu Ihrem Hauttyp:**

**Welche Farbe hat Ihre Haut (ohne Sonne)?**

 Rötlich

 Weißlich

 Leicht beige

 Bräunlich

**Hat Ihre Haut Sommersprossen?**

 Ja, viele

 Ja, einige

 Ja, aber vereinzelt

 Nein

**Wie reagiert Ihre Gesichtshaut auf Sonne?**

 Sehr empfindlich, meist Hautspannen

 Empfindlich, teilweise Hautspannen

 Normal empfindlich, nur selten Hautspannen

 Unempfindlich, ohne Hautspannen

**Wie lange können Sie im Frühsommer in Deutschland (Meeresspiegelhöhe) mittags bei wolkenlosem Himmel sonnenbaden, ohne einen Sonnenbrand zu bekommen?**

 Weniger als 15 Minuten

 Zwischen 15 und 25 Minuten

 Zwischen 25 und 40 Minuten

 Länger als 40 Minuten

**Wie reagiert Ihre Haut auf ein längeres Sonnenbad?**

 Stets mit einem Sonnenbrand

 Meist mit einem Sonnenbrand

 Oftmals mit einem Sonnenbrand

 Selten oder nie mit einem Sonnenbrand

**Was geschieht bei einem Sonnenbrand?**

 Kräftige Rötung, teilweise schmerzhaft und Bläschenbildung, danach schält sich die Haut

 Deutliche Rötung, danach schält sich die Haut meist

 Rötung, danach schält sich die Haut manchmal

 Fast nie Rötung und Hautschälen

**Kommt es bereits nach einem einmaligen, längeren Sonnenbad zu einer Bräunung?**

 Nie

 Meist nicht

 Oftmals

 Meist

**Wie geschieht nach wiederholtem Sonnenbaden?**

 Kaum oder gar keine Bräunung

 Leichte Bräunung nach mehreren Sonnenbädern

 Fortschreitende, deutlicher werdende Bräunung

 Schnell einsetzende und tiefe Bräunung

**Welche Haarfarbe haben (oder hatten) Sie?**

 Rot bis rötlich braun

 Hellblond bis blond

 Dunkelblond bis braun

 Dunkelbraun bis schwarz

**Welche Augenfarbe haben Sie?**

 Hellblau, hellgrau oder hellgrün

 Blau, grau oder grün

 Hellbraun oder dunkelgrau

 Dunkelbraun
